# The Adaptive Immune System of *Haloferax volcanii*

**DOI:** 10.3390/life5010521

**Published:** 2015-02-16

**Authors:** Lisa-Katharina Maier, Mike Dyall-Smith, Anita Marchfelder

**Affiliations:** 1 Department of Biology II, Ulm University, 89069 Ulm, Germany; E-Mail: lisa-katharina.maier@uni-ulm.de; 2 School of Biomedical Sciences, Charles Sturt University, 2650 NSW, Australia; E-Mail: mike.dyallsmith@gmail.com

**Keywords:** CRISPR-Cas, PAM, crRNA, Cas6, Cascade, archaea, *Haloferax volcanii*, type I-B

## Abstract

To fight off invading genetic elements, prokaryotes have developed an elaborate defence system that is both adaptable and heritable—the CRISPR-Cas system (CRISPR is short for: clustered regularly interspaced short palindromic repeats and Cas: CRISPR associated). Comprised of proteins and multiple small RNAs, this prokaryotic defence system is present in 90% of archaeal and 40% of bacterial species, and enables foreign intruders to be eliminated in a sequence-specific manner. There are three major types (I–III) and at least 14 subtypes of this system, with only some of the subtypes having been analysed in detail, and many aspects of the defence reaction remaining to be elucidated. Few archaeal examples have so far been analysed. Here we summarize the characteristics of the CRISPR-Cas system of *Haloferax volcanii,* an extremely halophilic archaeon originally isolated from the Dead Sea. It carries a single CRISPR-Cas system of type I-B, with a Cascade like complex composed of Cas proteins Cas5, Cas6b and Cas7. Cas6b is essential for CRISPR RNA (crRNA) maturation but is otherwise not required for the defence reaction. A systematic search revealed that six protospacer adjacent motif (PAM) sequences are recognised by the *Haloferax* defence system. For successful invader recognition, a non-contiguous seed sequence of 10 base-pairs between the crRNA and the invader is required.

## 1. The CRISPR-Cas Immune System

**Figure 1:**
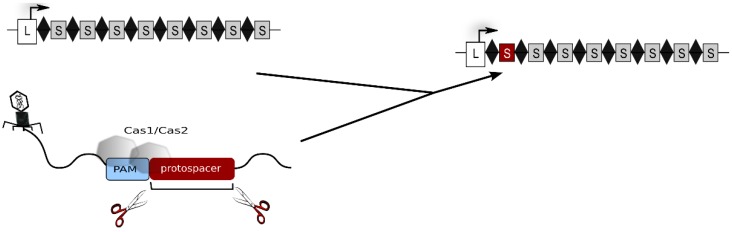
Acquisition of new spacers. The invader DNA is degraded by Cas proteins and a piece of the invader DNA is integrated as a new spacer (shown as red rectangle) into the CRISPR locus. Repeats are shown as diamonds, spacers as grey rectangles and the leader region as white rectangle. The leader is located at the 5' end of the CRISPR locus. The CRISPR locus including the novel spacer is shown at the right, the original CRISPR locus is shown at the left. The invader DNA to which Cas1 and Cas2 bind is shown at the bottom.

The CRISPR-Cas system is the most elaborate defence strategy present in prokaryotic cells (for general reviews about the CRISPR-Cas system see: [1,2,3,4,5,6,7,8,9]). It confers immunity against foreign genetic elements by a sequence-specific targeting and elimination of the invading nucleic acids. To this end, the cell establishes and maintains a genetic record of previously encountered viruses and plasmids within its CRISPR loci. These genomic regions are arrays of recurring repeat sequences, between which are short variable spacer sequences that represent genetic samples of invader DNA [10,11]. CRISPR loci not only provide genetically heritable systems for specific immunity but also, by their transcription, give rise to a key player of CRISPR defence, the crRNA. In proximity to CRISPR loci are gene cassettes encoding Cas proteins, that are responsible for all parts of the defence reaction: acquisition of foreign DNA (spacer sequences), crRNA biogenesis as well as target degradation. The defence reaction progresses in three stages. In the first stage, new spacer sequences are acquired. Here, as shown in Figure 1, a short piece of invader nucleic acid is selected and integrated into a CRISPR locus [5,7]. For this step, type I and II systems require short sequence motifs, called PAMs [12,13]. These motifs are part of the invader DNA and are used by the adaptation machinery for selecting the invader DNA fragment to be integrated. In addition, they are essential for the recognition and degradation of the invader upon a recurring infection.

The second stage of CRISPR activity covers the biogenesis of the crRNAs. CRISPR loci are transcribed into long precursor molecules, which are processed into much smaller, mature crRNAs, each containing a spacer sequence and parts of the flanking repeat sequences. The spacer sequences render each crRNA specific for a particular invader. The third stage, referred to as interference, occurs when the cell is invaded by intruder DNA. If the CRISPR locus contains a spacer sequence matching this invader (*i.e.*, captured from a previous invasion event), then the resulting crRNA will guide the CRISPR associated complex for antiviral defence (Cascade) complex to recognize the intruder DNA, which ultimately leads to degradation of the foreign nucleic acid via the activity of the protein components of these complexes [5].

CRISPR-Cas systems have been classified into three major types (I, II, III) [1] that can be further subdivided into 14 subtypes, each showing significant differences in the nature of their Cas proteins as well as mechanistic details of the defence reaction [1,14,15,16]. Subtype III-B systems are a clear example of this variation, as they target RNA, whereas all other currently known subtypes target DNA. To allow for a complete and comprehensive picture of this defence mechanism, it is essential to analyse all CRISPR-Cas systems in a variety of species. Since very good overviews about the CRISPR-Cas system and its function in general have been recently published [1,2,3,4,5,7,8,14,17], this review focuses on the type I-B system of *Haloferax volcanii*.

## 2. The Type I-B CRISPR-Cas System of *Haloferax volcanii*

**Figure 2:**
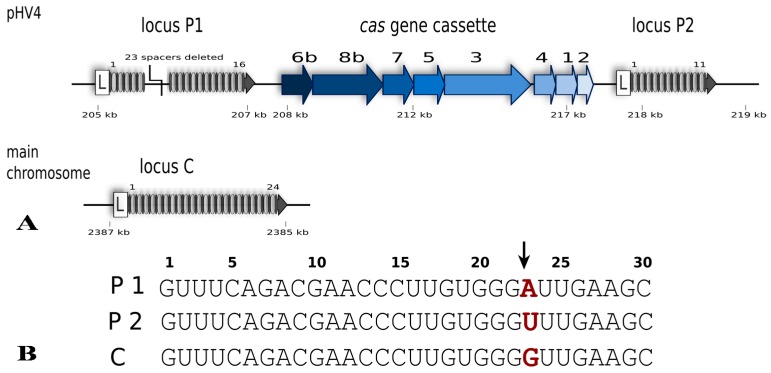
The CRISPR-Cas type I-B system of *Haloferax volcanii*. (**A**) The system consists of eight Cas proteins and three CRISPR arrays. Specific for class I systems is the presence of the Cas3 protein. The presence of a Cas8b protein defines this system as type I-B. The *cas* gene cluster is flanked by two of the CRISPR loci while the third locus is encoded on the main chromosome. In comparison to the published genome sequence of *Haloferax* strain DS2 [22] the H119 strain has a deletion in CRISPR locus P1 (23 spacers and repeats deleted) [20]. Gene locations on pHV4 and the main chromosome are indicated (in kb) but their sizes are not to scale. (**B**) The repeat sequences of the three CRISPR loci are identical except for one nucleotide at position 23 (shown in red). Processing of the CRISPR RNA by Cas6b takes place between nucleotides 22 and 23 in the repeat sequence (indicated by an arrow) leaving an 8 nucleotide repeat sequence upstream of the spacer and the remaining 22 nucleotides of the repeat downstream of the spacer.

*Hfx. volcanii* is a halophilic euryarchaeon first isolated from the shores of the Dead Sea [18]. It grows best at around 45 °C, requires a salinity of approximately 2.5 M NaCl and maintains an equally high intracellular salt concentration [18,19]. *Haloferax* possesses a single CRISPR-Cas system of subtype I-B, with three different CRISPR loci; one on the main chromosome (locus C) and two on the large (636 kb) chromosomal plasmid pHV4 (locus P1, P2) (Figure 2) [20,21]. The P1 and P2 loci flank the single *cas* gene cassette that carries genes for eight Cas proteins (Cas1-8b). The repeat sequences of all three CRISPR loci are 30 nt in length and identical in sequence (in all but one nucleotide), whereas spacer sequences vary in length from 34 to 39 nucleotides.

All three loci are actively transcribed and the transcripts processed, leading to a stable population of mature crRNAs [20]. In 2012, only two spacers of *Hfx. volcanii* (C-14 and P1-2) showed likely matches to sequences in the public databases [20], but this has now been considerably expanded (Table 1), and has revealed prominent types of invader DNAs. The C-14 spacer shows exact matches to the genomes of two recent isolates of *Haloferax*, and targets homologs of *Hfx. volcanii* gene HVO_0372 (Table 1). This ORF occurs in similar gene contexts in at least five different isolates of *Haloferax*, and appears to be within an integrative mobile element (Hvol-IV1) of ~12 kb, that commonly attacks members of this genus, most likely a temperate virus. In their integrated (provirus) state, they are flanked by a tRNA^Ala^ gene at one end (*att*L), and an integrase and partial copies of the tRNA (*att*R) at the other end (Supplementary Figure S1); a typical arrangement first described in temperate bacteriophages. The significance of this virus group (denoted as HFIV1) in the natural environment is highlighted by CRISPR spacers from other species that target the same virus: one from *Hfx. denitrificans* that targets the same gene but at a different position, and another from *Hfx*. sp. ATCC BAA-645 that targets a nearby gene (HVO_0375) (Table 1). Spacer C-4 closely matches a gene within a previously documented (defective) provirus of *Hrr. lacusprofundi*, Hlac-Pro1 [23], as well as related viruses in *Hfx. elongans* and* Hfx. mucosum* (denoted HeloV2 and HmucV2, respectively). These all show relationships to halovirus BJ1, an integrative virus of *Halorubrum*, but HeloV2 and HmucV2 differ significantly from BJ1 in not carrying integrase or tRNA genes, and both appear (from the available sequence data) to exist in cells as circular plasmids (Supplementary Figure S2). Spacer P1-2 matches a sequence within *Htg. jeotgali* ORF HL44_04258, encoding a conserved ParBc (plasmid partition) domain containing protein. The closest known homologs of this protein (and many other ORFs around it and elsewhere on the same contig) are bacterial or phage/plasmid related, indicating a region of mobile foreign DNA. Other spacers match metagenomic sequences from salt lakes (P1-3, P2-1), including one that targets a MCM (helicase) gene. Finally, the P2-11 spacer exactly matches CRISPR spacers found in three other species of *Haloferax* that were isolated in different countries (Spain, Israel and Egypt), indicating a significant and widespread invading element, and presumably a preference or selective advantage for the retention of this particular protospacer. In summary, the matches discovered so far are consistent with the spacers of *Hfx. volcanii* representing sequences recovered from invader (foreign) DNA, such as viruses and plasmids.

**Table 1:** Sequences closely similar or exactly matching CRISPR spacers of *Hfx. volcanii* DS2.

Spacer	Alignment of spacer/matching sequence^a^	Matching sequence
**C-4** (nt 2386433:2386398)	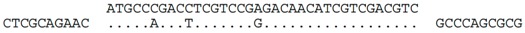	*Hrr. lacusprofundi* chromosome 1 (nt 759433:759468), within ORF Hlac_0754. Predicted translation of spacer is identical to protein Hlac_0754 but for one conservative (L/F) change (*i.e.*, MPDLVRDNIVDV/MPDFVRDNIVDV). Hlac_0754 is part of a 28.7 kb region (nt 750728:779675) containing genes related to halovirus BJ1, and previously denoted by Krupovic *et al*. [23] as provirus Hlac-Pro1.
**C-4** (nt 2386433:2386398)	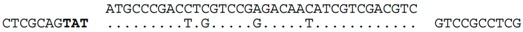	*Hfx. elongans* ATCC BAA-1513: AOLK01000020. Within ORF C453_12906 (nt 35718:35683), a homolog of Hlac_0754. Predicted translation of spacer exactly matches the protein sequence of C453_12906 (MPDLVRDNIVDV). Contig AOLK01000020 is likely to represent a halovirus genome, with many genes related to BJ1 or other haloviruses/plasmids. We denote this contig as HeloV2. A related virus appears to be represented by a contig (AOLN01000009) of *Haloferax mucosum* PA12, ATCC BAA-1512 (*i.e.*, HmucV2, Supplementary Figure S2)
**C-14** (nt 2385742:2385778)	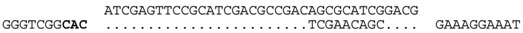	^b^ *Hfx. volcanii* (chromosome, nt 333928:333984), within ORF HVO_0372 (hypothetical protein). HVO_0372 occurs in a ~12kb region of foreign DNA flanked at one end by a tRNA-ala, and at the other end by an integrase and two partial repeats of the tRNA-ala gene. This region appears to be a provirus (we denote as Hvol-IV1). Related provirues are found in the genomes of at least four other *Haloferax* species (see Supplementary Figure S1). A nearby gene, HVO_0375 (CPxCG-related zinc finger protein), is the likely target of a CRISPR spacer of Hfx. sp. ATCC BAA-645 (contig_24).
**C-14** (nt 2385742:2385778)	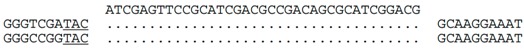	Line 2: *Haloferax* sp. ATB1 (JPES01000108.1) scaffold108 (nt 9103: 9067). Line 3: *Haloferax* sp. BAB2207: ANPG01000768 (nt 1559–1596). Both matches occur within a homolog of HVO_0372, and are likely to be part of proviruses (Hatb-IV1 and Hbab-IV1, see Figure S1). Elsewhere in this gene (and in HVO_0372) is a target sequence matching a CRISPR spacer carried by *Hfx.denitrificans ^d^*.
**P1-2** (nt 205072:205108)	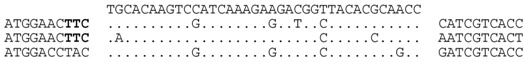	Line 2: Lake Tyrrell metagenome (contig 1101968716470, library GS84-02-2-3kb, nt 851:887) ^b^. Line 3: metavirome (assembly from SRR402046). Line 4: *Haloterrigena jeotgali* A29, HL44_contig00019.19 (nt 18864:18136), within locus tag HL44_04258. BLASTX predicts a COG1475 (ParBc domain) protein (plasmid partition protein). Closest relatives are bacterial (e.g., WP_021624091). The predicted aa sequences are identical *i.e.*, HKSIKEDGYTQP.
**P1-3** (nt 205139:205173)	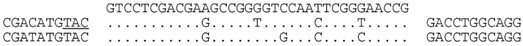	Line 2: Lake Tyrrell metagenome (49037 1101497529448, library GS84-02-2-3kb, nt 190:224). Line 3: metavirome (assembly from SRR402046). BLASTX of matching contigs show matches to Hbor_29150 of *Hgm. borinquense*. The adjacent gene, Hbor_29160, on the genome most closely matches M201_gp84 of halovirus HCTV-2. The predicted aa sequences over the matching region (left) are identical but for one conservative change (F/L), *i.e.*, VLDEAGVQFGNR / VLDEAGVQLGNR
**P1-38** (nt 207450:207485)	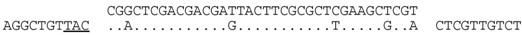	Lake Tyrrell metavirome (assembly from SRR402046). BLASTX of matching contig shows a match (E = 10^−13^) to the integrase of halovirus HCTV-5 (M200_gp113). The predicted aa sequences of spacer and matching contig sequence differ by one conservative (D/E) change *i.e.*, RLDDDYFALEAR/RLDDEYFALEAR.
**P2-1** (nt 217843:217879)	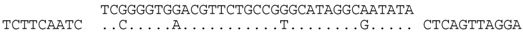	Great Salt Lake metagenome sequence 162854 GSLNARP_GFPJP1N02GIUFX (nt 107:73). BLASTX shows strong similarity (E = 10^−24^) to MCM/cdc46 family proteins (e.g., *Natrialba taiwanensis* (ELY91445), and the spacer sequence translates to an identical aa sequence, *i.e.*, YIAYARQNVHP to that of the matching GSL sequence.
**P2-2** (nt 217911:217945)	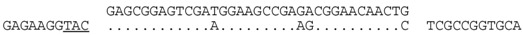	*Haloferax* sp. ATB1: ATB1DRAFT_JPES01000050_1.50(nt 15622:15655). Match occurs within a hypothetical protein gene (ATB1DRAFT_01921), with homologs in other species, e.g., *Hfx. mucosum* (ELZ94997.1), *Hfx. mediterranei* (AFK19012.1) and *Hgm. borinquense* Hbor_29150 (see P1-3, above). The predicted aa sequence of spacer and matching genome are similar, *i.e.*, AESMEAETEQL/AESKEAEAEQL.
**P2-10** (nt 218436:218471)	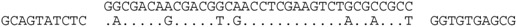	*Haloferax* sp. ATB1: ATB1DRAFT_JPES01000088_1.88, nt 33483:33448. Matches a surface glycoprotein-like ORF (ATB1DRAFT_03295), with many haloarchaeal homologs (e.g., D320_03738, C456_06592 and HVO_2072).
**P2-11** (nt 218503:218535)	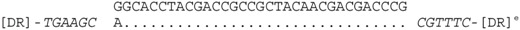	**CRISPR spacer** of * Haloferax lucentense* DSM 14919: AOLH01000022 (nt 8242–8273).
**P2-11** (nt 218503:218535)	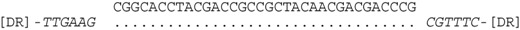	**CRISPR spacer** of *Haloferax* sp. BAB2207: ANPG01000305 (nt 8,276–8,244).
**P2-11** (nt 218503:218535)	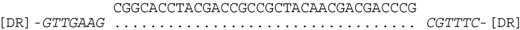	**CRISPR spacer** of * Haloferax alexandrinus* JCM 10717: AOLL01000024 (nt 43672–43639).

^a^ PAM motifs previously reported are in bold type. Underlined bases represent upstream motifs (at the same position as PAMs) that are frequently observed in database matches, but not found by *in vivo* experiments. Bases in italic font are alignments with other CRISPR spacers (*i.e.*, P2-11), and represent positions in the CRISPR repeat sequences. Dots in alignments represent bases identical to the spacer sequence above. ^b^ Sequence matches reported previously by Fischer *et al*. [20] ^c^ Alignment of *Hfx. sp.* ATCC BAA-645 CRISPR spacer to a sequence in HVO_0375 of *Hfx. volcanii*.

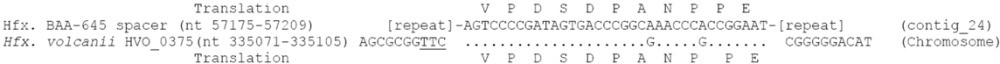
^d^ Alignment of *Hfx. denitrificans* CRISPR spacer to *Hfx. volcanii* (HVO_0372) and to Hfx. sp. ATB-1 (ATB1DRAFT_03991):

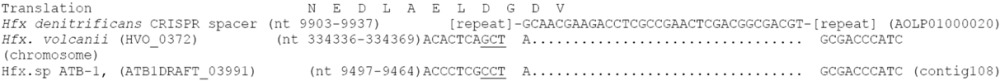
^e^ CRISPR repeats sequences flanking spacer sequences are shown in italic font.

Despite a clearer picture emerging regarding origins of eight spacers (Table 1), *Haloferax volcanii* carries a total of 74 spacers, so the targets of the great majority of spacers remain unsolved. This relatively low success rate could reflect one or more of the following possibilities: (a) that archaeal and especially haloarchaeal viruses are still underrepresented within the databases due to low levels of sampling; (b) the population of viruses and plasmids existing in the Dead Sea in 1975 (when *Hfx. volcanii* was isolated) may not be common (or exist at all) in the world now; and (c) that the viruses/plasmids represented in the *Hfx. volcanii* CRISPR loci are still present and widespread, but have evolved considerably over the 40 years since 1975.

## 3. Generation of crRNAs and Composition of the Interference Complex

Processing of the primary CRISPR RNA transcript (pre-crRNA) into functional crRNAs is a pivotal step of the defence activity. Each mature crRNA contains a spacer sequence flanked by repeat fragments at the 5' and 3' ends [24,25]. The spacer sequence promotes binding of the crRNA to Cas7 and mediates the sequence specificity of the defence system, enabling recognition by base-pairing to the invader DNA. The function of the repeat sequence is to bind to and position the cRNA on the Cascade complex [26,27,28].

In type I systems, the processing reaction is catalysed by the Cas6 endonuclease (the exception is the type I-C system, where this reaction is carried out by Cas5c [29,30]), cleaving the pre-crRNA upstream of the spacer to yield a crRNA with an eight nucleotide 5' handle and the remainder of the repeat at the 3' end [25]. Phylogenetic analyses reveal a tight evolutionary linkage between the repeat sequences and the Cas6 protein [31,32]. Although all Cas6 proteins catalyse the same reaction, they show wide differences in amino acid sequence, with only two motifs being common to all members of the Cas6 protein family: a glycine rich motif and a ferredoxin fold [33]. Biochemical analyses have shown that they even vary in the amino acids used for catalysis, and have different modes of binding to their RNA substrates [24,25]. In most type I systems, Cas6b is part of the Cascade-like complex [24,25,26,27,28].

**Figure 3:**
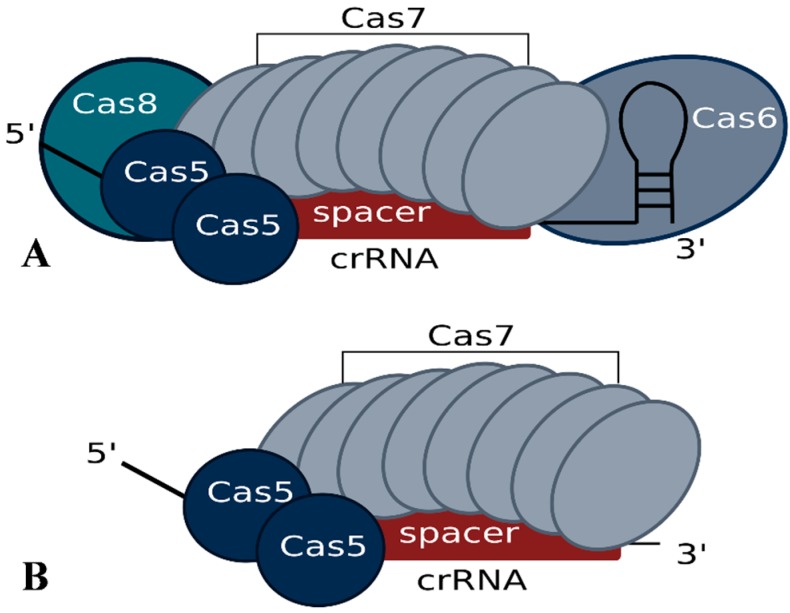
Potential Cascade complex composition of the *Haloferax* type I-B system modeled according to the published structure of the *E. coli* I-E system. Experimental data regarding the actual structure has not yet been reported. (**A**) According to the iBAQ analysis the Cascade complex in *Haloferax* contains 8.5 Cas7 proteins, 1 Cas6b and 1.7 Cas5 proteins. In addition we observed a loose association of Cas8b. (**B**) The minimal stable Cascade complex could consist of just Cas5 and Cas7 and the short crRNA. Cas8b is essential for the interference reaction but only loosely associated with the complex.

In *Haloferax*, deletion of the *cas6b* gene results in loss of crRNAs, confirming the essential role of the Cas6b protein in crRNA metabolism for the I-B system [34,35]. Further analysis showed that for a normal steady state concentration of crRNAs not only Cas6b but also Cas5 and Cas7 are required, suggesting that they protect the crRNA from degradation [34]. *Haloferax* cells with only Cas5, Cas6b and Cas8b (e.g., without Cas7) still contain crRNAs, but they are present at a significantly reduced level. While Cas8b can, to some extent, also account for crRNA level stabilization, crRNAs are most efficiently protected when Cas7 is present. A co-purification approach using FLAG-tagged Cas7 protein revealed that the Cascade-like complex of *Haloferax* contains Cas5, Cas6b and Cas7 subunits [34]. The Cas8b protein seems to be very loosely attached since it cannot be reproducibly co-purified, suggesting that the *Haloferax* I-B Cascade complex has a core of Cas5 and Cas7 and that Cas6b and Cas8b are more loosely associated (Figure 3). Using mass spectrometry and intensity-based absolute quantification (iBAQ) the components of the core complex were shown to occur in the following ratio 1.7:1:8.5 (Cas5:Cas6b:Cas7) [34]. This composition differs from the observed composition of the type I-E Cascade complex, which consists of 1 Cas5, 1 Cas6, 6 Cas7, 1 Cas8 and additionally contains two copies of the small subunit Cse2 [26,27,28]. The greater number of Cas7 proteins needed by *Haloferax* may be due to a difference in spacer length. In *Haloferax*, spacers are 34–39 nt in length whereas in *E. coli* they are only 32 nt. An additional Cas7 protein may be needed to cover the extra 2–7 nucleotides of spacer.

## 4. Characteristics of a Functional crRNA and Its Interaction with the Invader

**Figure 4:**
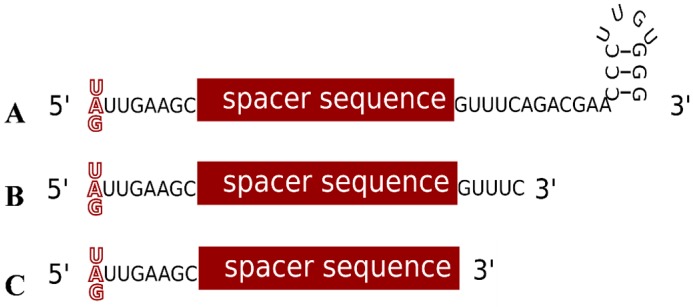
The different forms of the crRNA. (**A**) The long form of the crRNA found *in vivo* contains the spacer sequence, an eight nucleotide long 5' handle and a 22 nucleotide long 3' handle. (**B**) The short form of the crRNA found *in vivo* contains only five repeat derived nucleotides at the 3' end resulting in a shorter 3' handle. (**C**) The shortest functional version of the crRNA does not contain a 3' handle at all.

The central molecule of the defence reaction is the crRNA, consisting of spacer and repeat sequences [25]. The repeat sequences of the haloarchaeal CRISPR RNAs are highly conserved and can form a stem loop structure with a three bp stem (Figure 4A) [36]. Analysis of the *Haloferax* crRNA population by high-throughput sequencing revealed that cleavage of the pre-crRNA takes place between nucleotides 22 and 23, right at the base of the potential hairpin motif (Figure 2 and Figure 4) [36]. This leaves an eight-nucleotide 5' handle originating from the upstream repeat sequence (that precedes the spacer sequence), and a 22-nucleotide 3' handle downstream of the spacer. As mentioned above, the CRISPR repeats of *Haloferax* are identical except for one position, nucleotide 23 (Figure 2B), and after processing this would result in mature crRNAs that differ at the first base (the 5' nucleotide, Figure 4). RNAseq and northern blot analysis showed that the majority of the stably maintained crRNA population are between 64 and 69 nt in length (due to spacer length differences), with an average of 66 nucleotides [36]. The crRNAs consist of the eight nucleotide 5' handle, the 34–39 nt long spacer and the 22 nt long 3' handle. Further analysis revealed a second population of crRNAs with a 3' handle of only five nucleotides. This differs from crRNA maturation in other type I systems, where after the initial cleavage by Cas6 no further processing is observed (types I-A, I-E and I-F) [25]. A similar shortening of the 3' end has been reported for the type I-B systems in two other microorganisms, *Methanococcus maripaludis* and *Clostridium thermocellum* [35]. Together, these data clearly show that type I-B crRNA maturation is different from the same process in type I-A, -E and -F systems.

The requirements for a functional crRNA were able to be examined more closely after developing a Cas6 independent system for crRNA maturation, and then testing crRNA mutants for activity in interference [37]. It was shown that the crRNA 5' handle is critical for the interference reaction, whereas the 3' handle is dispensable. This agrees with *in vivo* observations of a shorter crRNA population that carries only a five nucleotide long 3' handle, and it will be important to determine whether the short form is the actual active form of the crRNA. Using the same experimental method, we could also show that the *Haloferax* CRISPR-Cas system does not depend on the chemical nature of the end groups of crRNAs, as the independently generated crRNAs possess a 5'-phosphate and 3'-hydroxyl group, whereas crRNAs generated by Cas6 cleavage result in a 5'-hydroxyl and a 3'-phosphate or 2'-3'- cyclic phosphate group, respectively [37].

Even though the primary CRISPR transcripts would be expected to lead to equimolar levels of mature crRNAs, the steady state levels of individual crRNAs in *Haloferax* have been found (by RNAseq) to differ [36], and this has also been seen in other systems [35,38,39,40,41,42]. This might be an artefact from biases occurring within the current RNAseq technology, or reflect true differences in crRNA stability due to variable nature of spacer sequences. For example, different spacer sequences could be more or less tightly bound by the Cascade proteins, and so alter their exposure to RNA degrading enzymes. Even if different crRNAs are present in equimolar proportions, they may not elicit equal defence reactions. In studies comparing the ability of different crRNAs to fend off the same plasmid invader, clear differences were seen in their efficacy [36]. No clear correlation could be found between the efficacy of particular crRNAs and their characteristics, such as their abundance, spacer length or sequence, G/C content, *etc.* Since the spacer segment of the crRNA directly interacts with the protein subunits of the Cascade complex, it could be that there are multiple factors that can interact and influence the microarchitecture, electrostatic interactions and topology within the complex, and also affect interactions with the cognate DNA target.

A curious observation made in the *Haloferax* I-B system may indicate that the context of the target sequence in the invader is also an important factor for successful defence. It was found that the ability to fend off plasmid invaders was strongly dependent on the mode of plasmid replication [36]. Plasmids with a pHV1 origin of replication could be readily fended off, but plasmid invaders with a different origin of replication (pHV2) could not be degraded [36]. The pHV1 ori uses an origin recognition complex (ORC) based mode of replication that binds CDC6 [43,44] whereas the pHV2 ori is most likely Rep protein dependent [45,46]. Since the origin of replication and the invader (target) sequence are located directly next to each other in the plasmid constructs used in these studies, additional experiments are required to show whether the different modes of replication interfere sterically with the defence system or whether another interaction between the defence system and the replication machinery is behind this observation.

**Figure 5:**
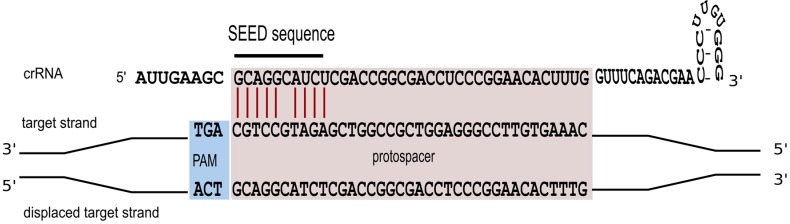
A seed interaction is required for effective interference. To efficiently target the invader the crRNA has to base pair with the invader sequence over a ten nucleotide non-contiguous sequence. Base paring at position six is not required. Essential base pairs are shown in red.

Triggering a defence reaction critically depends on base pairing between the spacer segment of the crRNA and the cognate protospacer region of the foreign invader [47]. To study characteristics of this interaction in more detail, a systematic mutagenesis of the protospacer sequence was carried out [36]. We could show that perfect base pairing within the first 10 nucleotides of the spacer sequence is an essential prerequisite for defence activity (Figure 5). Only position 6 is not required to base pair. A comparable seed-sequence is also found in *P. aeruginosa* and *E. coli* [47,48,49]. For *E. coli* the seed sequence is a seven nucleotide non-contiguous sequence that allows for a gap at position six [48]. The structural information now available for the *E. coli* Cascade complex [26,27,28] makes it immediately clear, why the sixth nucleotide cannot base pair with the invader. The thumb domain of the Cas7 contacts the crRNA at this position, kinking the RNA chain and causing this nucleotide to be flipped-out and point away from adjacent bases, so that it cannot take part in target binding.

## 5. Motifs for Detecting Invaders

The interference reaction not only depends on factors and features contained within the host cell but also on motifs found within the invader genome. Short sequence motifs flanking the protospacer region (PAMs) are needed in order to mount a defence reaction [1,12,13]. PAM sequences are found in type I and II CRISPR-Cas systems and play important roles in both protospacer selection in the adaptation process, as well as invader identification during the interference stage [13]. The PAM sequences for the *Haloferax* I-B system have been identified *in vivo* by systematically changing bases within the PAM and testing their effect in a plasmid based invader system [20,50,51]. Six PAM sequences (TTC, ACT, TAA, TAT, TAG, CAC) were shown to be effective in the *Haloferax* CRISPR-Cas system, the highest number of PAM sequences for any organism determined so far [17]. Since then, a high number of motifs triggering interference have been described in a couple of other organisms, and presumably offers a strategy for the host cell to cope with clonal divergence and individual mutations within the invader population [13]. This not only impedes escape by mutations within the PAM sequence but also broadens the recognition potential to include closely related foreign elements.

**Figure 6:**
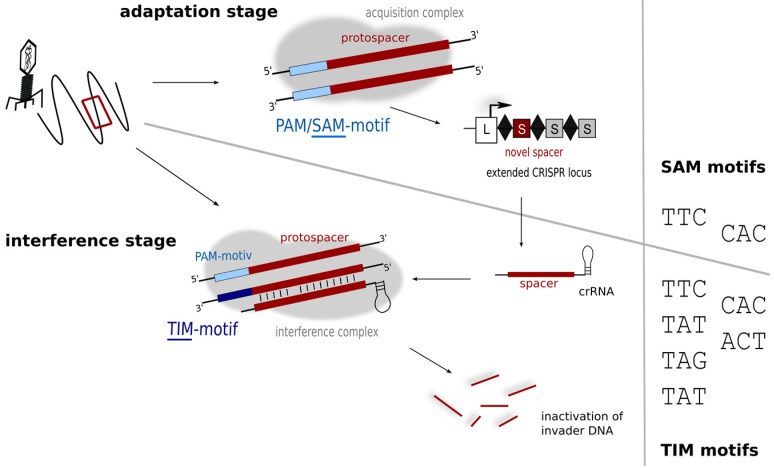
Motifs for protospacer acquisition and for target detection. In the adaptation step (upper panel) fewer motifs (SAM motifs) might be detected, while in the interference step more motifs are recognised and trigger degradation. Interference experiments using plasmid invaders show that six different motifs (TIMs) can trigger degradation in *Haloferax*.

While the plasmid invader approach was able to identify the PAMs used by *Haloferax* in the defence stage, it does not reveal the motifs driving the acquisition process, which can so far only be inferred from sequence alignment data (of cognate spacers and target invaders). If the sequences of the invader elements present in the Dead Sea in 1975 were known then this would be relatively easy to determine, but these data are not available, so the alignments presented in Table 1 must be interpreted with caution. Some matching sequences display PAMs that are consistent with laboratory findings (TAT, TTC, CAC; shown in bold type in Table 1) while others do not. One of the latter, TAC, appears to be overly represented. The diversity seen in the PAM region of the alignments likely reflects the diverse origins and nature of the matching sequences, mostly genomic/metagenomic data (rather than metaviromes). In contrast, an *in silico* comparison of *Haloquadratum walsbyi* (type I-B) spacers and metavirome sequences identified a number of likely sources of spacer acquisition events, and the associated PAMs were almost always the same: TTC [20,52]. PAM sequences are assumed to be connected to repeat sequence and CRISPR subtype [13], and given the high conservation of haloarchaeal repeats [36] and the presence of a subtype I-B system in both of these organisms, coincident PAM requirements seem reasonable. Hence, only a subset of PAM sequences linked to the defence reaction appear to be active in the acquisition step. Such a constraint and more stringent PAM usage has now been demonstrated in several other organisms representing different subtypes [13], which has led to a subdivision of PAM sequences into motifs important for acquisition of new spacers (SAM—spacer acquisition motif), and those essential for the interference reaction (TIM—target interference motif) (Figure 6) [13].

A general picture is emerging that suggests the number of SAMs is limited, whereas most organisms tend to tolerate a broader variety of TIMs [13]. As the interference reaction depends on the activity of the Cascade complex, interaction with the TIM motif would be probably with the Cas8b protein. However sensing of the SAM during spacer acquisition depends on a different subset of Cas proteins (probably Cas1 and/or Cas2) details of this interaction are not known yet since the process of acquisition is not fully understood. The binding interfaces in these two phases may well differ in amino acid composition, and so might impose different demands for PAM sequences.

## 6. Selection Pressure for Retention of the *cas* Genes

We have shown that the *cas* gene cassette of *Haloferax* can easily be removed upon selective pressure, with no apparent effects on cell growth or viability [20]. However, the CRISPR-Cas system has been retained in laboratory strains of this organism that have been grown in pure culture for 30 years. This raises the question of whether the CRISPR-Cas system might exert effects within the *Haloferax* cell that make it indispensable even in the absence of selective pressure by invading foreign genetic elements.

## 7. Conclusions

Taken together, the characteristics of the type I-B system of *Haloferax volcanii* summarised here are, on one hand, in agreement with the features described for other type I systems, but they also confirm that clear differences exist between subtypes. To trigger degradation, a PAM sequence flanking the targeted invader sequence is required, as is the case in other class I subtypes. The Cascade-like complex present in *Haloferax* is also similar to analysed examples of the type I-E system in that a seed sequence is required between crRNA and invader DNA for the interference reaction. However, in contrast to other type I systems, the crRNAs of the type I-B system of *Haloferax* undergo an additional maturation step, which occurs after cleavage by Cas6, and produces a shorter crRNA. This has also been observed in other type I-B systems [35]. This shorter crRNA cannot bind Cas6, as it does not contain the part needed for this interaction, and so crRNA binding to Cascade [34] results in a complex that does not include Cas6. This example of a significant mechanistic difference between CRISPR-Cas subtypes highlights the importance of analysing all the subtypes, and across a range of different organisms, in order to achieve a comprehensive understanding of this remarkable immune system and its diversity.

## Supplementary Files

Supplementary File 1
