# Supplementary material for: The Adaptive Immune System of Haloferax volcanii
Source: Life (Basel). 2015 Feb 16;5(1):521–37. doi: 10.3390/life5010521 (PMC4390866; doi:10.3390/life5010521)
Supplement: Supplementary File 1 [file life-05-00521-s001.docx]

Supplementary


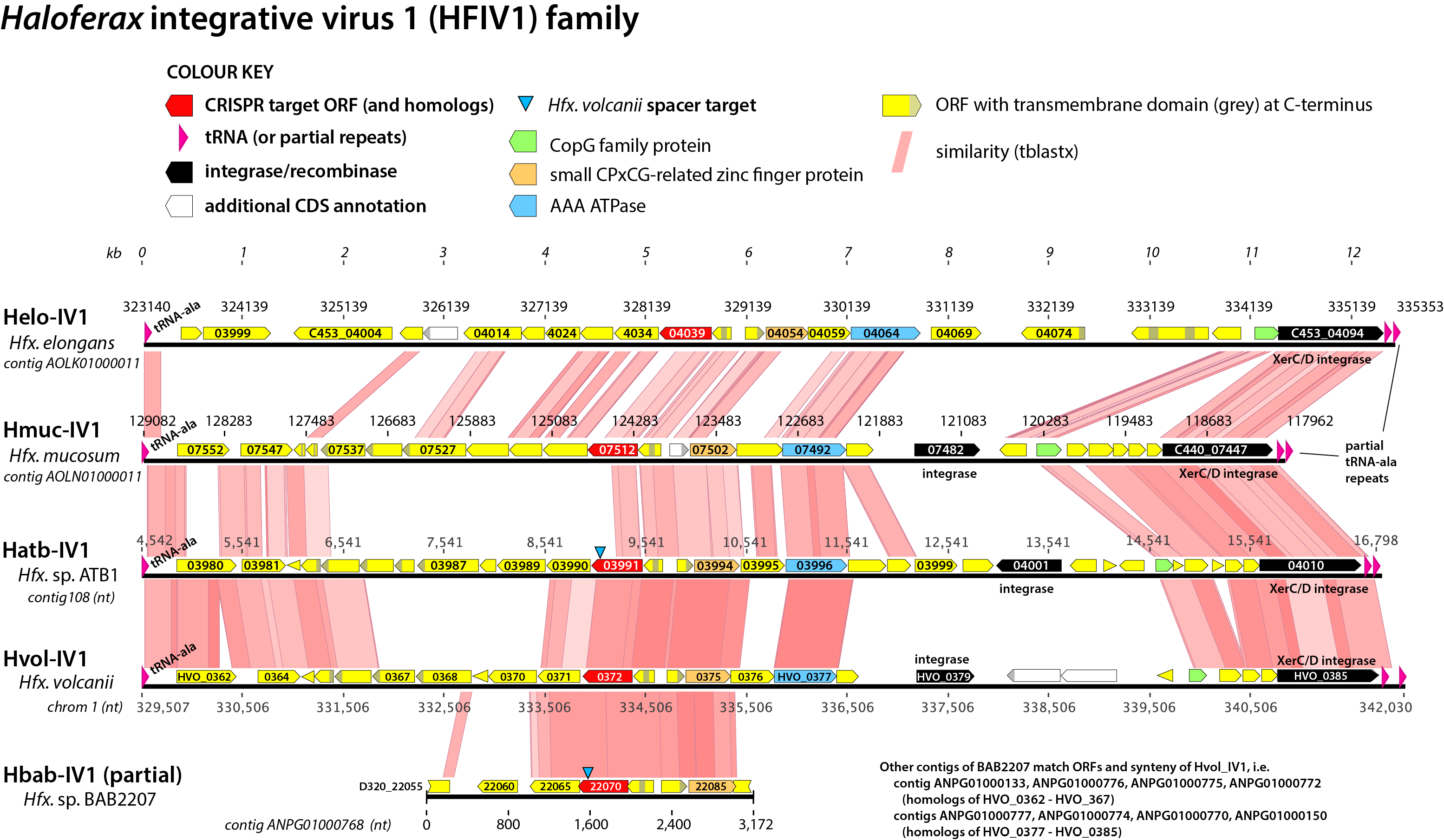


**Figure S1.** *Haloferax* integrative virus 1(HFIV1) family. All members of this group appear to integrate into a tRNA gene (*att*B), producing a short duplication at the other end (*att*R). Close inspection of the *att*R region revealed a second short repeat of the tRNA-ala (possibly representing two *att*P sites). All members appear to carry a XerC/D integrase/recombinase at the right end, consistent with temperate bacteriophages carrying *att*P sequences that allow them to integrate into the tRNA genes of their hosts. Regions of sequence similarity between members were determined using the Artemis comparison tool (Version 13.0.0, Wellcome Trust Sanger Institute: Hinxton, UK) with the tblastx option, and are shown in the diagram as pink bars connecting vertically between the genomes. ORFs are shown as blocks with arrows indicating their orientation. Contig names shown at the left of each sequence, and nucleotide (nt) positions in these contigs are shown just above the ORF boxes (with a scale just below the colour key, in kb). Locus tags are given in ORF boxes where there is sufficient space. Vertical blue arrows show where the CRISPR spacer C-14 of *Hfx*. *volcanii* matches a sequence found in the corresponding ORFs of *Hfx*. sp. ATB1 and *Hfx*. sp. BAB2207, both homologs of HVO_0372. Currently (December 2014), the *Hfx*. sp. BAB2207 genome remains as many short contigs, but similarity searches revealed other contigs of this genome that would give a gene pattern similar to that of the other examples shown above it. The HVO_0372 ORF and its homologs in the other genomes are coloured red. The colour key on top indicates other features of the diagram, such as transmembrane (TM) domains in protein sequence (which also show strong conservation among homologous proteins). Similarity and synteny between members appears to be greatest in the central region and at the ends. Very few ORFs have database matches or conserved protein domains that give clues to their function (a common finding in previously described haloarchaeal viruses).


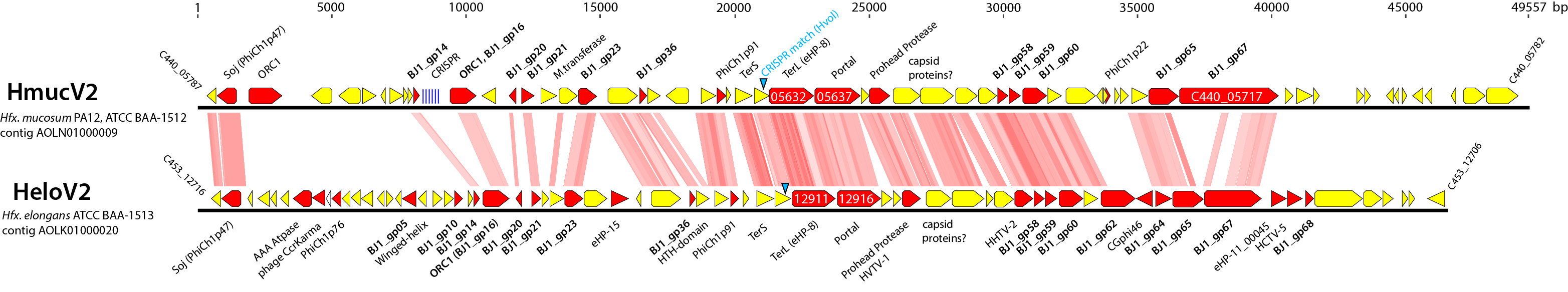


**Figure S2.** Comparison of putative temperate haloviruses HeloV2 and HmucV2. The genome sequences for *Hfx*. *mucosum* (PA12, ATCC BAA-1512) and and *Hfx*. *elongans* (ATCC BAA-1513) were downloaded from Genbank, and the contigs containing CRISPR matches to spacer C-4 identified. Contig AOLN01000009 (Hfx. mucosum) and AOLK01000020 (*Hfx*. *elongans*) had redundant sequences at their contig ends, and these were found to represent the ends of a single (broken) CDS. From this, it appeared that both contigs represented circular plasmids, so the redundant ends were trimmed and the ends joined to form circles. These were opened at a position that allowed the synteny to halovirus BJ1 to be best illustrated. ORFs are shown as blocks with arrows indicating their orientation. Locus tags for the initial and final CDS are given at the left and right ends, respectively, and a scale is shown above the contigs (in bp). Sequence similarity between the HmucV2 and HeloV2 was determined using the Artemis Comparison tool (ACT; tblastx option), and are shown as light pink strands between the two sequences. The sites of the matching sequence to Hfx. Volcanii CRISPR spacer C-4 are indicated by vertical blue triangles. These occur within a CDS immediately upstream of TerL. The putative proteins encoded on both contigs were checked for similarity by BLASTP (and by reference to Krupovic *et al*. [1]) and the resulting descriptions are given above and below the ORFs of each virus. Homologs of halovirus BJ1 proteins are indicated by bold type, and they follow the same gene order (synteny); from BJ1_gp05 at the left to BJ1_gp68 at the right, and there are many genes that match those of other haloviruses or bacteriophages (Terminase, Portal, prohead protease). Similarity to virus homologs other than BJ1 are also given, e.g., haloviruses PhiCh1 (*Natrialba*; NC_004084.1), HCTV-5 (*Haloarcula*; NC_021327), environmental halophage eHP-8 (JQ807229.1), *etc*.). HmucV2 appears to carry part of a CRISPR locus (blue vertical lines), indicating that these viruses have the ability to capture host DNA. Both carry orc1/cdc6 genes, presumably enabling them to replicate as plasmids.

Reference

1. Krupovic, M.; Forterre, P.; Bamford, D.H. Comparative Analysis of the Mosaic Genomes of Tailed Archaeal Viruses and Proviruses Suggests Common Themes for Virion Architecture and Assembly with Tailed Viruses of Bacteria. *J. Mol. Biol.* **2010**, *397*, 144–160.
